# Role of AKT/mTORC1 pathway in pancreatic β-cell proliferation.

**Published:** 2012-09-30

**Authors:** Norman Balcazar Morales, Cecilia Aguilar de Plata

**Affiliations:** a Facultad de Medicina, Universidad de Antioquia. E-mail: nobamo@gmail.com; b Facultad de Salud, Universidad del Valle. E-mail: caplataa@yahoo.es

**Keywords:** Diabetes mellitus, type 2, islets of langerhans, signal transductions, cell proliferation, AKT/PKB/mTORC1signaling pathway, Cell cycle

## Abstract

Growth factors, insulin signaling and nutrients are important regulators of β-cell mass and function. The events linking these signals to regulation of β-cell mass are not completely understood. Recent findings indicate that mTOR pathway integrates signals from growth factors and nutrients with transcription, translation, cell size, cytoskeleton remodeling and mitochondrial metabolism. mTOR is a part of two distinct complexes; mTORC1 and mTORC2. The mammalian TORC1 is sensitive to rapamycin and contains Raptor, deptor, PRAS40 and the G protein β-subunit-like protein (GβL). mTORC1 activates key regulators of protein translation; ribosomal S6 kinase (S6K) and eukaryote initiation factor 4E-binding protein 1.

This review summarizes current findings about the role of AKT/mTORC1 signaling in regulation of pancreatic β cell mass and proliferation. mTORC1 is a major regulator of β-cell cycle progression by modulation of cyclins D2, D3 and cdk4/cyclin D activity. These studies uncovered key novel pathways controlling cell cycle progression in β-cells in vivo. This information can be used to develop alternative approaches to expand β-cell mass in vivo and in vitro without the risk of oncogenic transformation. The acquisition of such knowledge is critical for the design of improved therapeutic strategies for the treatment and cure of diabetes as well as to understand the effects of mTOR inhibitors in β-cell function.

## Abstract (es)

Factores de crecimiento y nutrientes son reguladores muy importantes de la masa y función de las células β, pero las vías de señalización que unen estas señales a estos procesos no han sido completamente elucidadas. Estudios recientes han demostrado que la proteína mTOR integra señales provenientes de factores de crecimiento y disponibilidad de nutrientes con procesos celulares como transcripción, traducción, organización del citoesqueleto y metabolismo mitocondrial. mTOR puede hacer parte de dos complejos diferentes, mTORC1 y mTORC2. En el complejo mTORC1, la proteina mTOR la cual es sensible a rapamicina y se encuentra asociada a las proteínas Raptor, G β L, deptor y PRAS40, activa reguladores claves en la síntesis de proteínas, tales como la proteína cinasa ribosomal S6 (S6K) y la proteína de unión al factor eucariótico de iniciación 4E.

El presente trabajo recopila información reciente sobre la participación de la vía de señalización AKT/mTORC1 en la regulación de la proliferación y masa de las células β del páncreas. mTORC1 regula la progresión del ciclo celular en células β, mediante la modulación de los niveles de las ciclinas D2 y D3 y la actividad del complejo Cdk4/ ciclina D. Estos estudios que revelan nuevos puntos de control del ciclo celular en células β, pueden ser utilizados en el desarrollo de nuevos enfoques para expandir la masa de células β, sin el riesgo de inducir una transformación oncogénica. Los resultados relacionados en el presente trabajo aportan información muy valiosa para el desarrollo de nuevas estrategias terapéuticas para el tratamiento la diabetes tipo 2.

## Introduction

Diabetes mellitus type 2 (DM2) occurs when the β-cells of the pancreas cannot secrete sufficient amounts of insulin to meet the metabolic demand generated by the resistance to the effect of insulin in tissues such as muscle, liver and adipose tissue. Numerous studies have shown that insulin resistance precedes the development of hyperglycemia in individuals who eventually develop type 2 diabetes. However, there is a period in which blood glucose levels are almost normal, a situation attributable to β-cells compensating for insulin resistance by increasing its secretion either by increasing its mass, thereby synthesizing and secreting more insulin, or a combination of both mechanisms.

The ability of β-cells to increase their mass in response to insulin resistance is a critical factor in maintaining glucose homeostasisand preventing the development of DM2. β-cell mass is regulated by a dynamic balance of formation of new cells from progenitor (neogenesis), proliferation (duplication from other pre-existing), variation in cell size and apoptosis^1^. Experiments using genetic lineage marking β-cells showed that replication is the main mechanism for the maintenance of β-cell mass in adult mice^2^. This mechanism was confirmed by experiments measuring the short and long-term uptake of 5-bromo-2-deoxiurinida (BrdU) in the β-cells of adults mice^3^. It was also observed that growth factors(e.g. insulin and IGF-1), incretins, glucose and amino acids regulate β-cell mass by stimulating cell proliferation. Although the molecular mechanisms that induce proliferation are diverse, many studies have shown that the signaling pathway of insulin receptor substrate-2 (IRS-2) /phosphoinositol-3-kinase (PI3K)/AKT/PKB plays a critical role in the regulation of the mass and function of pancreatic β cells^4^.

The participation of IRS2 in the regulation of mass and function of pancreatic β-cells was demonstrated in transgenic knock-out mice to which two alleles of the gene encoding this protein were eliminated. These animals developed severe diabetes, due to the inability of β-cells to expand in response to a state of resistance to insulin^5^. As mentioned above, though activation of IRS2 can stimulate two different signaling pathways (Ras/Raf and PI3K/AKT), it has been shown that the pathway AKT/PKB plays an essential role in the survival and proliferation of pancreatic β-cells.

## Signaling pathway AKT/PKB and cell cycle control in pancreatic β-cells.

**Figure 1.**
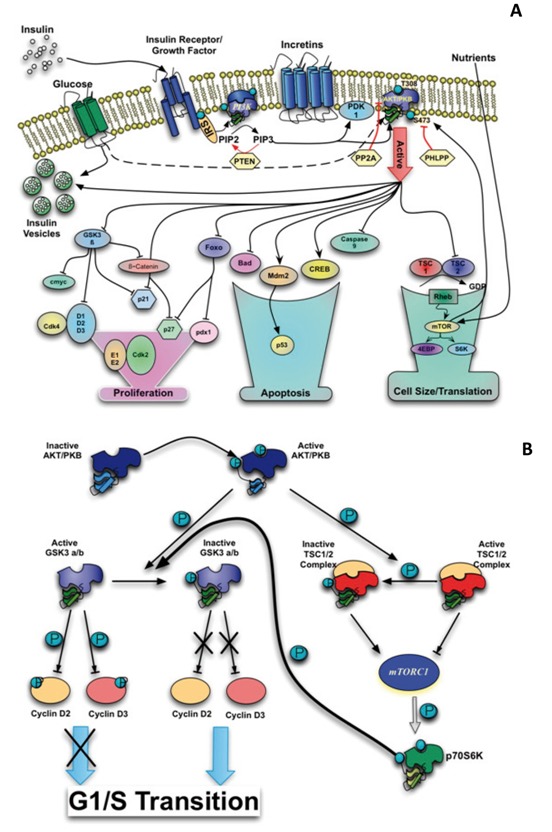
A. Schematic representation of different intracellular events involved in the signaling pathway AKT/PKB. B. Proposed regulation model of the levels of cyclin D2 and D3 by the mTORC1

Different studies suggest that the signaling pathway controlled by AKT/PKB not only plays an important role in insulin resistance but also in the ability of β-cells to adapt to an increase in insulin demand. The activity of AKT/PKB is regulated by mechanisms that are dependent and independent of PI3K- and require multiple steps involving its translocation to the plasma membrane and subsequent phosphorylation. The activation of AKT/PKB leads to phosphorylation of several substrates which control different signaling cascades, such as glucose transport mediated by insulin, glycogen and protein synthesis, cell growth, differentiation and survival^6^ (Fig. 1A).

In transgenic mice that overexpress a constitutively active variant of AKT/PKB in β-cells, an increase in proliferation, neogenesis, cell size, and synthesis-secretion of insulin, provide additional evidence of the involvement of this protein in the physiology of the pancreatic islets^7^ . In contrast, transgenic mice expressing a mutated version of this protein in their β-cells which produces an 80% reduction in kinase activity, glucose intolerance was observed owing to a defect in the secretion of insulin^8^.

The transition G1 → S of the cell cycle depends on the activity of different complexes of cyclin-dependent kinases (Cdk4/6) which are positively regulated by the levels of the cyclins D (D1, D2 and D3) and negatively by the level of their inhibitors (CIP: p21, p27 and p57 and INK4, p15, p16, p18, p19). Advances in the understanding of the mechanisms involved in cell cycle regulation have been obtained from mice deficient in Cdk4. These mice show growth retardation and diabetes. The development of diabetes is due to reduced β-cell mass during the first 6 weeks of life^9^. By contrast, mice expressing a Cdk4 mutation that prevents binding to INK4 inhibitors, show pancreatic hyperplasia due to abnormal β-cell proliferation^9^.

The role of cyclins D1 and D2 in β-cells was demonstrated in mice lacking the genes encoding these proteins. Mice deficient in the gene encoding cyclin D2 shows reduced β-cell mass and hyperglycemia within three months of age. The mice that additionally lack one allele of the gene for cyclin D1, have diabetes at 16 days of birth^10^. The levels of D-type cyclins can be regulated post-transcriptionally. In vitro studies showed that GSK3 β protein regulates cyclin D1 degradation through its phosphorylation on threonine 286^11^. Similar results have been obtained in experiments studying the stability regulation of the of cyclins D3^12^ and D2^13^. In the signaling pathway of insulin, GSK3 is phosphorylated and inactivated by AKT, suggesting that inhibition of GSK3 may be responsible for the stabilization of D-type cyclins through the activation of the PI3K/AKT pathway (Fig. 1A).

Studies in mice constitutively overexpressing active AKT protein in pancreatic islets (caAKTtg) showed that AKT induces hypertrophy, hyperplasia and hyperinsulinemia. To elucidate the molecular mechanisms involved in the regulation of β-cell mass of the pancreas this way, Fatrai *et al*.^14^, assessed the expression levels of cell cycle components regulating Cdk4 activity in both homozygous caAKTtg mice as well in caAKTtg mice that lacked one or both alleles of Cdk4. The results of these experiments suggest that AKT induces β-cell proliferation in a Cdk4-dependent manner and that increased levels of cyclin D1, cyclin D2 and p21 are associated with the proliferative response induced by the activation of AKT in these cells.

Levels and cellular localization of cell cycle inhibitors such as p21 and p27 also regulate the activity of Cdk4 and cell cycle progression. Phosphorylation of p27 by AKT, causes cytoplasmic retention, ubiquitination and degradation of this inhibidor^15^ (Fig. 1A). From studies in transgenic mice that overexpress or are deficient in p27 it is concluded that this inhibitor contributes to β-cells failure during the development of diabetes in mice that are insulin resistant^16^. Studies in mice that overexpressed p27 suggest that the role of p27 is critical during embryonic development of pancreatic β-cells since in the first 4 weeks of postnatal life these animals are glucose intolerant and have decreased proliferation and cell mass. By contrast, overexpression of this protein in adult mice has no effect on either glucose tolerance or in β-cell mass^17^. The phenotype identified in adult mice can be explained to be due to the low replicative rate and of cell renewal in adult animals. All these results indicate that the cyclin D/Cdk4 complex is an essential regulator in the proliferation of pancreatic β-cells.

mTOR, an important regulator of the size and cell proliferation. The protein TOR (Target of rapamycin) was identified in studies of genetic screening in yeast which sought to find the target of this antibiotic with potent immunosuppressive and anti-tumoral properties^18^. Rapamycin is an antifungal agent purified from a strain of *Streptomyces hygroscopicus* isolated from soil samples from Rapa Nui, an island in the South Pacific. It binds to the protein gene product FRP1 and forms a complex that inhibits TOR activity and cell growth. This mechanism is conserved in eukaryotes and orthologous genes have been identified in *C. elegans* ^19^, *Drosophila* sp^20^ and in mammals^21^. In these animals, rapamycin forms a complex with FK506 binding protein (FKBP) to inhibit mTOR (also known as FRAP, RAFT1 and RAPT1). Since its identification in yeast, multiple studies have established that TOR plays a central role in regulating cell size and proliferation.

mTOR can be part of two different complexes: mTORC1 and mTORC2^22^ ^,^ ^23^. In the complex mTORC1, it is bound to Raptor (Regulatory associated protein of mTOR), PRAS40, deptor and G β L (G protein subunit-like protein β, also known as mLST8). This form is sensitive to inactivation by rapamycin and regulates cell size through the activation of ribosomal S6 protein kinase (S6K1) and the inactivation of protein elongation factor binding of the polypeptide chain, eIF4E (4E-BP). These proteins regulate protein synthesis and ribosomal biogenesis^23^ ^,^ ^24^. Biochemical studies have shown that Raptor serves as a scaffold protein that facilitates contact between mTOR and mTORC1 substrates^24^. Deptor, present in both mTORC1 mTORC2 functions as an inhibitor of both complexes^25^. On the other hand, to PRAS40 has been assigned the role as mTORC1 inhibitor although its regulatory capacity has not been well defined.In the complex mTORC2, mTOR also joining G β L and deptor but also binds to rictor (rapamycin insensitive companion of mTOR) and to mSin1 and PRR5/protor^26^. 

Rictor and mSin1 promote the assembly and activation regulated by mTORC2 signaling. The role of PRR5/protor has not yet been determined. This complex is involved in regulating the remodeling of actin in the cytoskeleton and has recently been identified as the kinase that phosphorylates AKT at residue Ser473^27^. Unlike mTORC1, mTORC2 complex was judged as insensitive to inhibition by rapamycin but recent studies indicate that prolonged treatment with this drug can also inhibit mTORC2 activity in certain cell types^28^.

## Regulation of mTORC1 activity

Because protein synthesis requires the consumption of considerable amounts of energy it is natural to think that growth and cell proliferation are highly coupled to the availability of nutrients and energy. In mammalian cells, the signals from growth factors, availability of nutrients and energy are transmitted to mTOR that integrates these signals to properly regulate cell growth and proliferation.

When the insulin receptor is activated PI3K protein kinase is activated and mobilized to the cell membrane. This enzyme generates phosphoinositol 3,4,5 triphosphate which acts as a second messenger binding to pleckstrin homology domains (PH) present in the protein kinase AKT/PKB and on the phosphoinositol-dependent protein kinase (PDK1). In the cell membrane, PDK1 phosphorylates and activates Thr308 residue AKT/PKB^6^.

One of the mechanism by which growth factors, nutrients and energy, regulate the activity of mTOR is through the tuberous sclerosis complex (TSC). The complex consists of two proteins, hamartin or TSC1 (the protein product of gene tsc1) and tuberin or TSC2 (the protein product of gene tsc2). The heterodimer TSC2/TSC1 has an GTPase activation function on Rheb (Ras homolog enriched in brain)^29^. As a complex bound to GTP, Rheb is required for activation of mTORC1 and may exert its effect by binding directly to mTOR^30^. TSC2 phosphorylation by AKT inhibits the GTPase activity of the TSC2/TSC1 heterodimer allowing the Rheb-GTP complex to bind and activate mTOR (Fig. 1A).

Recent studies have revealed an alternative mechanism of regulation where AKT directly activates mTORC1 independently of TSC2/TSC1 complex. Immune-precipitation of mTOR followed by analysis by mass spectrometry allowed identification of the protein PRAS40 (proline-rich AKT/PKB substrate 40 kDa)^31^. PRAS binds and inhibits mTORC1 activity especially under conditions of fasting and energy stress but, when growth factors activate protein kinase AKT/PKB, this latter phosphorylates and exposes a recognition site for 14-3-3 on PRAS40. The 14-3-3 protein then dissociates from mTORC1 and PRAS40 get release of inhibition. One aspect to consider is that although PRAS40 has a predominant inhibitory effect on the activation-mediated inactivation of TSC1/ TSC2, PRAS40 deletion is not enough to activate mTORC1^31^.

Although activation of mTORC1 is mediated by intracellular signals triggered by growth factors these are not sufficient to stimulate the kinase activity of mTORC1 but instead is essential the availability of nutrients and energy. 

The properties of TOR proteins to detect the availability of nutrients have been evolutionarily conserved from yeast to mammals. In yeast, TOR senses changes in the availability of nitrogen, amino acids and glucose. Blocking the activity of TOR in yeast, through genetic mutations or treatment with rapamycin, results in a phenotype that resembles that obtained under fasting conditions^32^. In *Drosophila*, the deletion of TOR causes larval growth arrest a state resembling amino acid fasting^33^. Similar changes are observed in mTOR when cultured cells are subjected to glucose or amino acid fasting^34^.

Amino acids fasting, especially the absence of leucine and glutamine inhibits the phosphorylation of the effector proteins mTORC1, S6K and 4E-BP^32^. The interesting thing is that this inhibition occurs even in cells with deletion of the gene tsc2 when one would expect over-stimulation of mTORC1 activity. This inhibition will be reversed after the addition of medium with these amino acids. However, overexpression of Rheb reverses the inhibitory role of fasting amino acid and its inhibition using RNAi especially intensifies the inhibition of mTORC1^35^. These studies suggest that the amino acids regulation does not require TSC2, but the presence of Rheb.

It is not known the mechanism by which amino acids regulate the activity of mTORC1 by Rheb. Some authors suggest that the presence of amino acids may regulate the activity of a GTP exchange factor specific for Rheb or another GTPase-activating protein different toTSC2 but despite all that more studies are needed to understand the mechanism. Using biochemical, molecular and pharmacological approaches, it has been shown that a PI3K class 3, the hVps34 (human vacuolar protein sorting-34) is modulated by amino acid availability. The activation of S6K1 by amino acids through mTORC1 requires the hVps34 activation^35^. It is not known yet if hVps34 exerts its effect on Rheb or acts directly on mTORC1.

mTORC1 also transmits information about the cell's energy state. Reduced cellular ATP levels inhibit mTORC1 dependent cell signaling^36^. One mechanism by which mTORC1 detects energetic changes in the cell is through AMP-activated protein kinase (AMPK). AMPK is activated in response to increases in cAMP levels like it takes place in situations with high ATP turnover during periods of high metabolic demand. AMPK negatively regulates mTORC1 and prevents cell growth and proliferation during periods of energy shortage^37^.

On the other hand, AMPK activates TSC2 by phosphorylation of Thr1227 and Ser1345 residues. This activation leads to an increase in the hydrolysis of GTP bound to Rheb inhibiting its action on mTORC1^38^. The regulation of cellular energy sources can also be a regulator of mTORC1. Recent studies suggest that AKT increases energy metabolism by increasing intracellular ATP levels and inhibiting AMPK. In addition to the phosphorylation and direct inhibition on TSC2, AKT can inhibit TSC2 through inhibition of AMPK^39^. AKT-mediated mechanisms involved in maintaining high levels of ATP, partially imply, the maintenance of a high intake of nutrients^40^.

## mTORC1 downstream targets

One of the most studied target proteins of mTORC1 has been S6K1. mTORC1 activates S6K1 by phosphorylation on Thr389 and Ser371^41^ and this in turn phosphorylates and activates protein S6 of the ribosomal 40S subunit. When S6 is activated increases the association to the polysomic mRNA molecules with oligopirimidine sequences in their 5'-ends (TOP)^42^. The TOP motif is downstream of the 5'-cap of the mature mRNA and is present mainly in messengers encoding ribosomal proteins and elongation factors characteristic of protein synthesis^43^. Because of its role in the regulation of mRNA translation of ribosomal proteins, activation of S6K1/S6 is related to an increase in the cellular capacity for protein synthesis. However, recent studies have established the possibility of regulation of mRNA with TOPs, independent of S6K^44^.

Mice lacking S6K1 are smaller but survive normally without any detectable defect in cell size or in the regulation of proliferación^45^. However, mice lacking both homologous genes of S6K, S6K1 and S6K2 respectivelly, show perinatal lethality. The interesting thing is that cells from these animals have defects in cell cycle progression probably due to the fact that S6 can be phosphorylated by protein kinase mitogen-dependent p90rsk^44^. These studies demonstrate the redundancy in the function of S6K and p90rsk in the mitogen-mediated phosphorylation of S6. The smaller size found in *Drosophila* mutants deficient in S6K is endorsed to an effect on cell size rather than on cell number^46^.

Another target protein is the of mTORC1 binding protein elongation factor 4E (4E-BP1) which influences protein translation because it acts as translational repressor^47^. In cells deficient in nutrients or growth factors, 4E BP1 binds to the eukaryotic initiation factor eIF4E preventing its binding to the 5'-cap mRNA or the initiation factor 4G (eIF4G). The clear implication is that the complex formed by the mRNA, the initiation factors of translation and the 40s ribosomal subunit are not assembled and the translation is not performed. Phosphorylation of 4EBP by mTORC1 at multiple sites (Tre37/26, Ser64, Tre70) destabilizes its interaction with eIF4E allowing the binding of factors eIF4E/G and the formation of the initiation of translation complex. Thus, mTORC1 regulates the 5'-cap-dependent translation of transcripts.

In cells expressing a mutated form of 4EBP1 that cannot be phosphorylated by mTORC1, it remains bound to eIF4E and prevents translation of the mRNA resulting in decreased cell size. Additionally, overexpression of this mutant inhibits cellular cycle progression^48^. Consistent with this, overexpression of eIF4E rescues cells treated with rapamycin of decreased size and inhibition of cell cycle progression. On the other hand, cell lines that overexpress eIF4E lose control of cell division (development of malignant transformation) indicating that eIF4E is an important factor in regulating cellular proliferation^49^. Genetic studies in *Drosophila* showed that 4EBP1 mainly influences cell growth and has a less pronounced effect on proliferación^50^. These results indicate that 4EBP1 has important effects on growth and cell proliferation and its effect depends on the type and cellular environment.

## The role of mTORC1 in the proliferation of pancreatic β cells

Studies in pancreatic islets of mice and humans have shown that glucose activates mTORC1 and this activation, which is dependent on the availability of amino acids, stimulates cellular proliferation^51^. These authors suggest that the role of glucose and amino acids in the activation of mTORC1 is mediated by an increase in mitochondrial metabolism. Recent findings in MIN6 cells (mouse insulinoma cells) seem to support this idea. The availability of glucose and amino acids such as leucine and glutamine, increased ATP production by inhibiting the activation of AMPK and consequently activating mTORC1^52^.

The evidence of the importance of mTORC1 activation in the modulation of proliferation and its effect on β-cell mass comes from genetically modified mice. S6K-deficient mice with mutated versions of the S6 ribosomal protein have reduced β-cell mass, hypoinsulinemia and glucose intolerance^53^. To verify the role of mTORC1 as one of the key regulators of cell cycle progression and cell mass in pancreatic β-cells, it has been carried out a series of studies in genetically modified mice that activate mTORC1 signaling pathway. Rachdi *et al*.^54^ obtained mice with conditional deletion in the gene for tsc2 in pancreatic β-cells by crossing mice with the tsc2 gene flanked by the loxP sequence with mice expressing the cre recombinase under the control of the insulin promoter^55^. These mice had hypoglycemia, hyperinsulinemia, and improved glucose tolerance that was maintained through week 52 of life. The best prove of glucose tolerance was because of an increase in proliferation and cell size. These morphological findings were reversed by inhibition of mTORC1 by rapamycin treatment. The results suggest that mTORC1 regulates the proliferative signals induced by deletion of tsc2 gene in β-cells. Similar results were obtained in an animal model with a conditional deletion of the tsc1 gene. However, unlike animals with deletion of tsc2, these mice developed obesity and insulin resistance and did not show an increase in β-cell proliferation^56^. It is likely that despite acting as a complex, TSC1 and TSC2 show functional differencies. Another possibility to explain the difference in results would be that the mice in the two studies had different cre recombinase gene under the control of insulin promoter.

Using a doxycycline-inducible model (Tet-On), Balcazar *et al* ^57^studied the effects of mTORC1 inhibition under proliferative conditions induced by controlled activation of AKT. This animal model allowed the induction of cell proliferation and the increase of β-cell mass without altering peripheral tissue. The results of this study demonstrated that mTORC1 is a key component in regulating the cell cycle and this function is exerted through the activation of Cdk4. When AKT is overexpressed by using the doxycycline-inducible model, it reproduced some of the results found when using the model of constitutive overexpression of AKT (ca-AKTtg), although some differences were observed. For example, in the inducible model no changes were detected in cyclin D1 and p21 but in cyclin D3. Why the difference in the levels of expression of cyclins D and p21 between the models? One possible explanation would be that in the constitutive expression pattern AKT is expressed as soon as the progenitor cells differentiate to β-cells and start to produce insulin. Thus, the exposure time of these cells to overexpression of AKT is longer when compared with the inducible system and, at the time of the analysis of cell cycle components; the molecular scenarios are completely different.

The interesting and novel, is that changes in cyclin D2 and D3 detected in the inducible model are dependent on mTORC1 activity. These studies demonstrated for the first time that mTORC1 regulates the translation and stability of cyclin D2. Previously, it was shown that activation of GSK3 β/ β regulates ubiquitinationdependent proteosomal degradation of D-cyclins. AKT phosphorylates and inactivates GSK3 β/ β and this may explain some of the highest levels of cyclin D, cdk4 increased activity and consequently increased cell proliferation. What is surprising is that according to the results of Balcazar *et al*.^57^, S6K more than GSK3 appears to be the kinase responsible for phosphorylation and inactivation of GSK β/ β, that in turn could explain the mechanism by which mTORC1 regulates the stability of cyclins D2 and D3 (Figure 1B). mTORC1 inhibition by rapamycin promotes expansion of β-cells in a model of insulin resistance suggesting that mTORC1 coordinates the adaptation of these cells to hyperglycemia and the development of diabetes type 2^58^.

These results reveal a critical role of mTORC1 in the metabolism of carbohydrates and the regulation of β-cell mass. Also, supports the concept that modulation of the activation of the mTORC1 complex can be a very important component in the adaptive response of β cells to insulin resistance and cell damage.

Studies in pancreatic islet transplantation have demonstrated that administration of rapamycin inhibits the restoration of the cells, reduces the adherence of the graft and impairs the function of transplanted β-cells. The latter effect appears to be mediated by other factors like decreased glucose transporters and amino acids^59^. A better understanding of the effects of rapamycin in the mass and β-cell function is important to increase the successful transplantation of such cells.

Previous work provided new evidence for the involvement of mTORC1 in the regulation of proliferation and β-cell mass in the pancreas. The molecular mechanisms and signaling pathways downstream of this complex have been evaluated recently. Using a mouse model that overexpressed S6K protein in pancreatic islets, Elghazi *et al.* ^60^ reported that these animals showed better tolerance test glucose due to increased insulin secretion. One of the most notable aspects of this work is that unlike animal models where the signaling pathway activated AKT/mTOR these animals had a β-cell mass similar to that of the controls. These findings could be partially explained as a result of an inhibition of the signaling pathway of IRS1/2 /AKT and to the induction of insulin resistance in these cells. This pathway is critical in controlling the proliferation and survival of pancreatic β-cells. In this study, the absence of hyperplasia was associated with an alteration in cell cycle progression due to decreased Cdk2 levels and increased p16 and p27 inhibitors. On the other hand, increased apoptosis was associated with a decrease in the inactivation of pro-apoptotic signals such as phosphorylation of GSK3 β/ β and FoxO1.

The difference in the proliferative phenotype between this model and those used in the controlled activation of mTOR in β-cells can be, to some extent, explained for reasons like the latter pathway may activate other survival pathways and its effect on the inhibition of signal-regulated IRS are less critical.

The increase in insulin secretion observed in animals with overexpression of S6K suggests that this protein modulates selected metabolic aspects in β-cell function although the negative feedback inhibition of IRS signaling pathway affects the progression of cell cycle as well as the deterioration of the survival signs. 

## Conclusions and prospects

It is well established that the signaling pathway IRS2/PI3K/AKT is a critical regulator of the mass and function of the pancreatic β-cells. Proteins such as FoxO1 and GSK3 β are very important components that mediate proliferative and survival signals in response to activation of AKT/PKB. The works refered on in this review broaden this knowledge by adding the pathway TSC2/mTOR as another important component in the regulation of proliferation and cell size. By this, it can be concluded that there is a predominant pathway downstream of AKT/PKB to regulate β-cell mass. One could state however, that each pathway contributes to the phenotype observed as a result of activation of AKT/PKB. Despite the information available so far it has not been possible to clarify the molecular mechanisms responsible for alterations in cell proliferation and cellular apoptosis triggered by different protein targets of AKT/PKB. Experiments in mice with conditional deletions in the different components of the signaling pathway of AKT/PKB might allow understand the importance of these components in the regulation of β-cell mass.

The protein mTOR was originally identified as an important regulator of cell proliferation in response to growth factors. Today, it is clear that mTOR, in addition to growth factors, acts as a central regulator that integrates biological stimuli such as nutrient availability, energy and oxygen, as a coordinator of cell proliferation and growth, and mitochondrial metabolism.

One of the major trials would be the validation of the results obtained in the inducible expression approach of AKT using the conditional deletion system of Tsc2 gene, specifically in relation to the post-transcriptional modulation of the levels of cyclin D2 and D3 as well as the activity of cdk4. The Tsc2 gene product has a more direct effect on mTORC1, and less oncogenic potential that AKT, and by corroborating such results we will obtain valuable information to develop new therapeutic strategies for the control and treatment of type 2 diabetes.

Although, the results presented are from experiments in mice without any metabolic disorder, it is clear that they provide new advances in the understanding of the molecular mechanisms that control the cell cycle and proliferation of pancreatic β-cells which are critical components to the maintenance of cell mass. Because of the role played by the quantity and quality of β-cells in the development of type 2 diabetes, the information is of great importance since it identifies molecules of signaling pathways susceptible to be altered with the purpose of increase cellular proliferation.

In other words, this information can be used in the development of new approaches to the expansion of β-cell mass in vivo and in vitro without the risk of oncogenic transformation. The acquisition of such knowledge is critical for designing effective therapeutic strategies for treatment and cure of diabetes as well as to understand the effects of mTOR inhibitors in β-cell function. A better understanding of the effects of rapamycin in the mass and β-cell function would be very important for the advancement and success in islet transplantation.
